# Eosinophil extracellular trap formation is closely associated with disease severity in chronic rhinosinusitis regardless of nasal polyp status

**DOI:** 10.1038/s41598-019-44627-z

**Published:** 2019-05-30

**Authors:** Chi Sang Hwang, Sang Chul Park, Hyung-Ju Cho, Dong-Joon Park, Joo-Heon Yoon, Chang-Hoon Kim

**Affiliations:** 1 Department of Otorhinolaryngology, Yonsei University Wonju College of Medicine, Wonju, South Korea; 2 Department of Medicine, Graduate School, Yonsei University College of Medicine, Seoul, South Korea; 3 Department of Otorhinolaryngology, Yonsei University College of Medicine, Seoul, South Korea; 4 The Airway Mucus Institute, Yonsei University College of Medicine, Severance Hospital, Seoul, South Korea

**Keywords:** Chronic inflammation, Immune cell death, Mucosal immunology

## Abstract

Chronic rhinosinusitis (CRS) is a heterogeneous inflammatory airway disease involving non-eosinophilic and eosinophilic phenotypes, which translate to various endotypes. Activated eosinophils and neutrophils are known to generate extracellular traps consisting of DNA and cytotoxic granule proteins. We sought to investigate the presence of eosinophil and neutrophil extracellular traps (EETs and NETs, respectively) in human CRS tissues and to clarify the associations with their clinical features. Nasal polyp (NP) or ethmoid tissue slides of 43 subjects from endoscopic sinus surgery for CRS were analysed. Quantitative analysis of EETs and NETs was performed by confocal microscopy using immunofluorescent staining. For correlation study, the presence of NPs, number of infiltrating tissue eosinophils, preoperative Lund–Mackay scores, and other comorbidities were analysed. EET formation was observed to varying degrees in all CRS groups and was correlated with the number of tissue eosinophils (r  =  0.83, p  < 0.001) regardless of the presence of NPs. Patients with more EETs demonstrated higher Lund–Mackay scores (r  =  0.51, p  = 0.009), blood eosinophilia (r  =  0.80, p  < 0.001), and decreased olfactory function (r  = −0.65, p  < 0.001). No correlation between the extent of EET formation and the presence of atopy or asthma was apparent. However, none of the CRS groups containing neutrophils formed NETs in this study. Eosinophilic CRS indicates the presence of EETs. Formation of EETs could have a role in clinical decision-making and prediction of treatment outcome of CRS, regardless of NP status.

## Introduction

Chronic rhinosinusitis (CRS) is a common and challenging inflammatory disease that involves non-eosinophilic and eosinophilic phenotypes^1–3^. It is known that the latter is typically marked by a greater disease burden with respect to risk for comorbidities and the likelihood of recurrence after surgical intervention^4,5^. Likewise, it is more probable that patients with tissue eosinophilia will have nasal polyps (NPs) compared to patients without tissue eosinophilia^6^. However, there are no standard methods for evaluating tissue eosinophilia due to diversity in geographic conditions and uneven distribution within a tissue^3,7^. Eosinophilic NPs also show a variety of inflammatory cells, such as neutrophils and macrophages, that may play a role in the tissue remodelling process in NPs^8^. Therefore, there are limitations to the subtyping of CRS or NPs based only on inflammatory cells.

A previous study revealed that neutrophils are able to form extracellular structures containing DNA and granule proteins, so-called neutrophil extracellular traps (NETs)^9^. These novel structures are formed by activated neutrophils and can kill invading pathogens before they reach host cells, a phenomenon which was at the time of the study recognized as the third antimicrobial function of neutrophils following phagocytosis and secretion of soluble antimicrobials. Other, recent evidence has indicated that activated eosinophils show similar extracellular structures, named eosinophil extracellular traps (EETs), which are able to bind and kill bacteria extracellularly^10,11^. Such extracellular nets are known to form in a reactive oxygen species (ROS)-dependent manner after priming with complement factor 5a, eotaxin, interferon-γ, interleukin (IL)-5, lipopolysaccharides, *Staphylococcus aureus (S aureus)*, or thymic stromal lymphopoietin^10,12,13^. Although the mechanism continues to be disputed, their generation has such a distinct pathway from suicidal cell death that such cytolysis could be renamed extracellular trap cell death, so-called ETosis^14,15^.

In the early days, extracellular DNA trap was studied in infectious or autoimmune disease models; however, recently, it was also identified in noninfectious models, such as allergic diseases in relation to EETs^11,16,17^. For the first time, researchers in Belgium and Japan described the presence of EETs in IL-5^+^ NP and eosinophilic sinus secretion, respectively, from human eosinophilic CRS (ECRS) samples^18,19^. These data suggested that EETs can play either a beneficial or harmful role in the field of ECRS, as their increased presence is observed at the site of epithelial barrier defects. EET formation was directly induced on exposure to *S aureus*, which in turn was able to entrap the bacteria and facilitate a role in the host’s defense against microbes^12^. However, compared to NETs, EETs contain smaller amounts of protease that contribute to the stability and high viscosity of eosinophilic mucin, in turn impairing their clearance by inflammatory cells and antibiotics^20^. As a consequence, EETs might cause long-lasting adhesive luminal surfaces as postmortem functions, resulting in further compromise of the barrier dysfunction, along with more bacterial aggregation and growth of biofilms and the potential for reinfection and chronicity of disease.

Although EETs in inflamed foci might be involved in the pathogenesis of ECRS, there is a lack of research to date regarding non-ECRS and the aim of clinical correlation. Therefore, the purpose of the present study was to explore the presence and distribution of EETs in eosinophilic and non-eosinophilic human CRS tissues and to clarify the correlations between their quantifiable level and clinical features in patients with CRS. Notably, we observed NETs as well as EETs in this study because CRS involves various pathomechanisms showing high heterogeneity even in patients with ECRS with a dominant T_H_2 profile. In addition, the presence of NETs in CRS tissues is not yet known.

## Methods

### Subjects

The diagnosis of CRS was based on patient history, nasal endoscopy, and computed tomography (CT) according to a 2012 European position paper on rhinosinusitis and nasal polyps^21^. To qualify for inclusion in this study, subjects were required to (1) be over 19 years of age, (2) be refractory to medical treatment and thus require bilateral endoscopic sinus surgery, and (3) have data from a minimum follow-up period of six months. None of the subjects had taken any form of local or systemic corticosteroid for at least four weeks prior to the operation. In addition, patients were excluded if they had a history of mucociliary disorder or immunocompromised status. The present study was approved by the institutional review board (IRB) of Yonsei University College of Medicine (IRB no. 4-2017-0408). Informed consent was obtained from all individual participants prior to enrolment in the present study. All procedures performed in studies involving human participants were conducted in accordance with the ethical standards of the institutional and/or national research committee, and with the 1964 Helsinki declaration and its later amendments, or with comparable ethical standards.

Classification of CRS was based on the NP and the Japanese Epidemiological Survey of Refractory Eosinophilic Chronic Rhinosinusitis (JESREC) scoring systems^4^. According to the JESREC scoring system, four categories of preoperative clinical features were used to assess ECRS, as follows: (1) bilateral disease sites were given a score of 3; (2) presence of NP was given a score of 2; (3) a dominant shadow of ethmoid sinus on a CT scan was given a score of 2; and (4) eosinophilia in peripheral blood was given a score of either 4 (>2% and ≤5%), 8 (>5% and ≤10%), or 10 (>10%). A JESREC score value higher than 11 points was determined to indicate ECRS. Patients in the ECRS group were stratified into three subgroups according to severity (neither A or B = mild, A or B = moderate, A and B = severe) by adding the refractory score, as follows: (A) blood eosinophilia (>5%) and ethmoid-dominant shadow in CT; (B) comorbidity of bronchial asthma, aspirin, or nonsteroidal anti-inflammatory drug intolerance. Finally, 43 nasal tissues samples and five control tissues were classified into four groups and analysed with clinical parameters. The clinical and demographic data of the patients are summarized in Table 1.

**Table 1:** Characterization of the study population.

Characteristic	Non-ECRSsNP (*n* = 11)	Non-ECRSwNP (*n* = 10)	ECRSwNP (*n* = 11)	ECRSsNP (*n* = 11)	Control (*n* = 5)
Mean age, years	51.0 ± 9.1	48.1 ± 14.6	40.6 ± 14.5	45.0 ± 14.1	28.2 ± 9.8
Sex, M/F	8/3	7/3	4/7	5/6	5/0
BMI, kg/m^2^	25.1 ± 3.1	25.0 ± 3.2	23.3 ± 3.0	22.0 ± 1.8	23.7 ± 2.6
JESREC^†^	4.5 ± 2.1	7.4 ± 1.9	17.3 ± 1.3	15.9 ± 2.2	4.0 ± 0
LM score	11.5 ± 6.7	17.4 ± 4.5	19.2 ± 3.1	17.6 ± 5.6	0
KVSS II score	25.7 ± 7.6	13.8 ± 10.3	12.4 ± 8.8	19.2 ± 8.3	31.8 ± 2.6
IgE, KU/L	132.3 ± 93.2	47.0 ± 48.2	234.6 ± 153.9	165.3 ± 130.0	28.1 ± 21.5
Eosinophils in peripheral blood, %	1.9 ± 1.1	2.5 ± 1.4	10.9 ± 3.4	10.5 ± 3.6	2.2 ± 1.2
Allergy, −/+	6/5	6/4	0/11	1/10	4/1
Asthma, −/+	3/0	7/0	7/4	6/5	5/0
Aspirin (NSAIDs) intolerance, *n*	0	0	0	0	0

All continuous data are presented as mean ± standard deviation.

^†^The sum of JESREC score and refractory score.

Abbreviations: ECRS, eosinophilic chronic rhinosinusitis; NP, nasal polyp; ECRSsNP, ECRS without NPs; ECRSwNP, ECRS with NPs; BMI, body mass index; JESREC, Japanese Epidemiological Survey of Refractory Eosinophilic Chronic Rhinosinusitis; LM, Lund–Mackay; KVSS II, Korean Version of the Sniffin’ Stick test II; NSAIDs, nonsteroidal anti-inflammatory drugs.

### Sample collection

CRS biopsy tissues of 43 patients archived as 4% paraformaldehyde-fixed and paraffin-embedded samples obtained during routine functional endoscopic sinus surgery at Yonsei University Severance Hospital from January 2017 to December 2017 were analysed. Ethmoid tissues were obtained from CRS without NPs (*n* = 22), and NPs were obtained from CRS with NPs (*n* = 21). Additionally, inferior turbinates from five healthy subjects who were undergoing septal surgery because of anatomic deviations were collected as control tissues.

### Assessment of medical conditions

For the correlation study, preoperative taste and smell tests, preoperative Lund–Mackay CT score^22^, as well as number of tissue eosinophils, comorbidities, and surgical outcomes were assessed. Using the recently developed gustatory function test (YSK taste function test kit, RHICO medical Co., Seoul, Korea), the sum of recognition threshold for five tastants, including sweet, butter, salty, sour, and umami, was scored. Scores lower than 12 were considered to have hypogeusia, accordingly to previously described protocols^23^. Olfactory function test comprised the Korean version of Sniffin’ Stick II (KVSS II, Bughart, Wedel, Germany), and the sum of threshold, discrimination, and identification scores was defined as KVSS II score. KVSS II scores 0 to 20 were defined as “anosmia,” 20.25 to 27 as “hyposmia,” and 27.25 to 48 as “normosmia.” Such criteria were determined based on previous work^24^. Patients were considered to have allergy if the skin prick test was positive for at least one of the batteries of standardized aeroallergens in South Korea. Asthma was defined based on lung function analysis, including methacholine challenge testing.

### Immunofluorescent staining

EETs and NETs were identified as previously described^12,13,17^. EETs and NETs were visualized in 5-µm-thick sections of paraffin-embedded tissue slides by means of indirect immunofluorescence, followed by counterstaining for DNA. The sections were blocked by 1% BSA, 1% normal donkey serum, then incubated with anti-eosinophil cationic protein (ECP) polyclonal antibody (Biolegend, San Diego, CA) and anti-myeloperoxidase (MPO) polyclonal antibody (R&D Systems, Minneapolis, MN), respectively. Alexa Fluor 568 and Alexa Fluor 488 (both Invitrogen, Carlsbad, CA) were used for secondary incubation, respectively. Negative controls were incubated with nonimmune serum instead of primary antibody. DNA was visualized with 4′6-diamino-2-phenylindole (Invitrogen, Carlsbad, CA). After washing with phosphate-buffered saline, the slides were mounted in a drop of fluorescent mounting medium (DAKO, Glostrup, Denmark). Images were obtained with a confocal laser scanning microscope (LSM 700; Carl Zeiss Microimaging, Jena, Germany) and analysed using ZEN software (Carl Zeiss, Jena, Germany). EET-positive cells were identified by cells that produced extracellular, decondensed DNA in fibers, or in a web-like shape colocalized with ECP^11^. Eosinophils and EETs were counted in 10 high-power fields (HPFs) as previously described^16,17^, and values are expressed as mean ± standard error of the mean (SEM) per HPF throughout the article. In analogy to EET, NET was defined that extracellular DNA released by neutrophils in conjunction with MPO in a fashion similar to the structures previously described in patients with asthma^17^. All assessments of images processing were performed by two independent investigators in a blinded manner (C.S.H. and S.C.P.).

### Statistical analysis

For between-group comparisons, the Mann–Whitney *U* test or Kruskal–Wallis test with a two-tailed test was used. To describe the correlation between variables, linear correlation analysis was used to determine bivariate relationships with a Spearman correlation test. Data are expressed as mean ± SEM. All statistical analyses were conducted using the Statistical Package for the Social Sciences version 18.0 (IBM Corp., Armonk, NY). A p value < 0.05 was considered statistically significant.

## Results

### All groups had eosinophils that were found to form eosinophil extracellular traps in tissues

**Figure 1:**
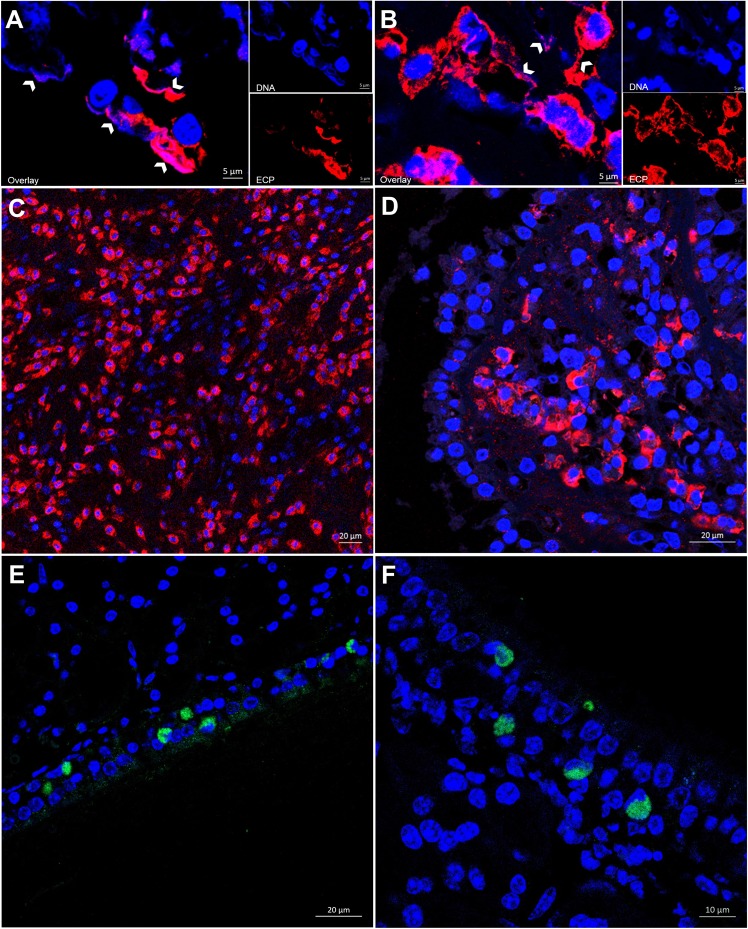
Representative image of endoscopic sinus surgery specimens from CRS subjects showing EETs. (**A**) The extracellular DNA was colocalized with ECP (indicated by the arrowheads) in the non-ECRS without NPs group. *Scale bar* = 5 µm. (**B**) In the case of ECRS with NPs, EET formation was more prominent (indicated by the arrowheads). *Scale bar* = 5 µm. (**C**) For the ECRS group, eosinophils located deep in the stroma were found to have mostly intact granules, and EET formation was very rare. (**D**) EETs were found mainly in the subepithelial regions, with clusters of eosinophils underneath the epithelial barrier defect. *Scale bar* = 20 µm. However, none of the groups were found to form NETs, even with the increased presence and alignment of neutrophils in the non-ECRS with NPs (**E**) or in the non-ECRS without NPs (**F**) groups. *Scale bar* = 20 µm.

EET-releasing eosinophils were observed in both the ECRS and non-ECRS groups. The confocal microscopy images clearly showed the presence of extracellular DNA nets in association with the granular protein ECP in the non-ECRS group (Fig. 1A). In the ECRS group, EET formation was more prominent (Fig. 1B). The numbers of EET-releasing eosinophils were highest in the apical part of the subepithelial region compared with those in the basal part or deep in the stroma. For the ECRS group, in the basal parts of the epithelium or deep in stroma, eosinophils were found to be mostly intact, and EET formation was very rare (Fig. 1C), whereas in the subepithelial regions with single-cell layers and missing epithelial cells, eosinophils were often seen in clusters and were often released as massive and clustered EETs (Fig. 1D). However, none of the CRS groups containing neutrophils formed NETs in this study. Even in the non-ECRS tissues, where a high number of neutrophils exceeding the number of eosinophils was observed, NETs were not found in the subepithelial region or the stroma (Fig. 1E,F).

**Figure 2:**
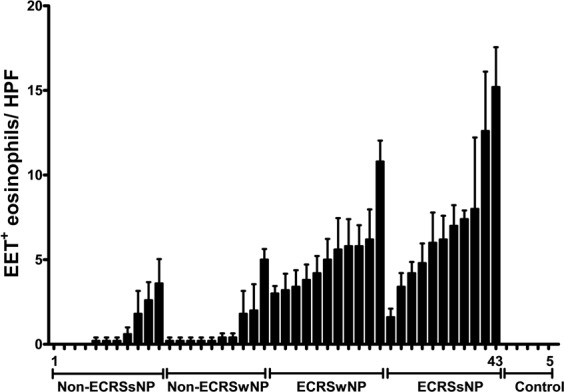
Column bar graph illustrating that EET-releasing eosinophils could be found in all CRS subtypes (subjects 1–43) but not in healthy control tissues. The proportion of eosinophils releasing EETs was significantly higher in ECRS group than in non-ECRS group. However, the presence of NPs exhibited no significant differences between CRS groups, which indicates that patients with ECRS may be more easily activated to produce EETs regardless of NP status.

With respect to the quantifiable level of ECP-DNA complex in each individual CRS subtype, Fig. 2 shows a mean value of EET-releasing eosinophils per HPF. EETs were expressed as a mean value of eosinophils generating EETs relative to the total amount of those present in 10 HPFs. No EET-releasing eosinophils were found in the healthy control group.

### Formation of eosinophil extracellular traps correlated with the number of tissue eosinophils regardless of the presence of nasal polyps

**Figure 3:**
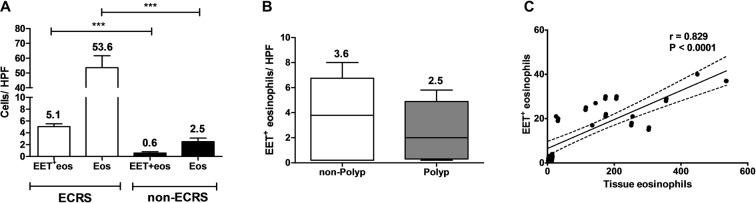
Quantitative analysis of infiltrating eosinophils and EETs according to CRS subtype. (**A**) The number of EET-releasing eosinophils and the total number of tissue eosinophils were significantly higher in the ECRS group than in the non-ECRS group. (**B**) However, the presence of NPs was not associated with EET formation. (**C**) Linear regression analysis between the numbers of EET-forming and total infiltrating eosinophils showed a statistically significant positive correlation. The lines indicate the mean with 95% confidence interval.

All surgical samples selected in this study were stained with haematoxylin and eosin. Eosinophils were observed rarely in the tissue of the control subjects. Although there were substantial differences between subjects, the ECRS group overall was infiltrated with a significantly higher number of tissue eosinophils than the non-ECRS group (53.6 ± 8.0 vs. 2.5 ± 0.6, p < 0.0001; Fig. E1). Moreover, the ECRS group demonstrated a significantly increased number of EET-releasing eosinophils versus the non-ECRS group (5.1 ± 0.5 vs. 0.6 ± 0.2, p < 0.0001; Fig. 3A). However, interestingly, EET formation was not associated with presence of polyps (3.6 ± 1.1 vs. 2.5 ± 0.6, p = 0.402; Fig. 3B), though EETs were correlated with total number of tissue eosinophils (r = 0.829, p < 0.0001, Fig. 3C). Considering all subjects, the mean fraction of EET-releasing eosinophils was 13.5% ± 8.8% (range: 0–35%) of the total amount of eosinophils infiltrating the nasal mucosa epithelium.

### Eosinophil extracellular traps appear to reflect activated eosinophils with a greater disease burden

**Figure 4:**
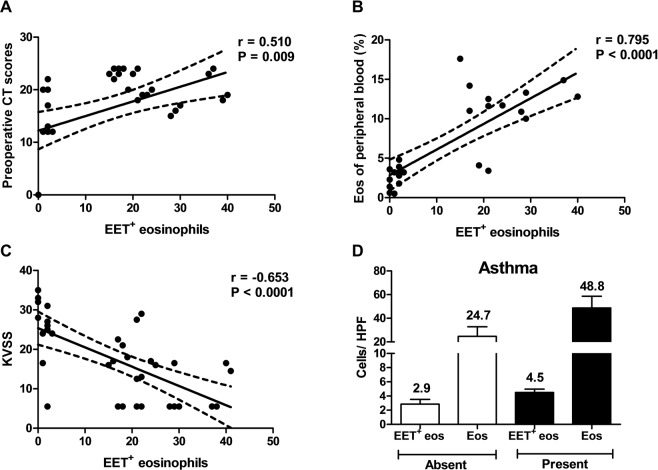
The extent of EET formation is correlated with (**A**) preoperative CT score, (**B**) decreased olfactory function, and (**C**) proportion of peripheral blood eosinophils, (**D**) but not with presence of asthma. The lines indicate the mean with 95% confidence interval.

EET-releasing eosinophils showed correlations with some clinical features in patients with CRS. We observed a higher number of infiltrating EET-releasing eosinophils in CRS specimens from subjects with a significantly high preoperative total Lund–Mackay CT score and a high proportion of peripheral blood eosinophils (r = 0.510, p = 0.009 and r = 0.795, p < 0.0001; Fig. 4A,B, respectively). In particular, a significant negative correlation was observed between the level of olfactory function and the EET-releasing eosinophils (r = −0.653, p < 0.0001; Fig. 4C). The number of tissue-infiltrating eosinophils and EET-releasing eosinophils tended to be higher in patients with comorbid of bronchial asthma compared to those without; however, the difference was not statistically significant (total number of tissue eosinophils: 48.8 ± 9.9 vs. 24.7 ± 8.1, p = 0.186, EET-releasing eosinophils: 4.5 ± 0.5 vs. 2.9 ± 0.7, p = 0.257; Fig. 4D). None of the other clinical variables including the taste test results and postoperative endoscopic findings were significantly different with regard to the number of EET-releasing eosinophils in this study.

## Discussion

The application of precision medicine in the field of CRS can be more effective after establishing the endotype. This is because CRS involves a diverse spectrum of inflammatory diseases showing high heterogeneity, which causes different therapeutic responses. Although eosinophil activation in inflamed foci is involved in the pathogenesis of ECRS, the factors that perpetuate the inflammation are not completely understood.

The present study demonstrates that some eosinophils in the inflamed nasal mucosa in both ECRS and non-ECRS patients form EETs, which consist of released filamentous chromatin structures in conjunction with eosinophil granule proteins in the extracellular matrix. In the group analysis, the ECRS group had a significantly higher presence of EET-releasing eosinophils than did the non-ECRS group, and unsurprisingly, EET formation was more often seen when the tissue eosinophils appeared more heavily extended. However, the presence of NPs was not a key component for the quantitative difference in EETs. This was a striking finding, since previous publications examined only the tissues or secretions from patients with ECRS with NPs^12,18,19,25,26^. In contrast to CRS subjects, none of the control biopsy specimens expressed EETs. EETs were observed mainly in association with disruption of the mucosal interface, which may indirectly confirm the findings of previous studies^12,16^ which reported that extracellular polymers are released as a net, similar to a spider-web, and trap invaded bacteria, thus participating in the host’s defense against microbes. Taken together, these findings, despite the lack of evidence of biological investigations, suggest that EETs might play a role in barrier function of the nasal mucosa via inflammation response in the pathogenesis of CRS, but are not directly related to the tissue remodelling process in NPs.

Possible trigger for the formation of EET in our non-ECRS group remains unclear. Given the subclassification of CRS by only using NP status and several preoperative clinical features in this study, it was difficult to simplify and dichotomize heterogeneous inflammation patterns in the comparison between ECRS and non-ECRS group. One recent study in the United States depicted that even CRS without NP is comprised of extremely diversity of cytokine patterns that 23%, 36%, and 15% of them showed T_H_1-, T_H_2-, and T_H_17-type inflammation, respectively, which indicate the overall frequency of type 2 inflammation is higher than type 1 inflammation^27^. In our study, ECRS group evidently had higher number of EET^+^ eosinophils, while also having high number of EET^−^eosinophils. Compared to non-ECRS, the ratio of EET^+^/ total eosinophils appears lower (5.1/ 53.6 vs. 0.6/ 2.5) in ECRS group, although there was no statistically significant difference. Within the limitation of this study, we assume that more activated status in eosinophils could be an essential part of the formation of EET rather than simply tissue eosinophil counts. This effect was observed in both ECRS and non-ECRS groups, with the majority of EETs being focused primarily at the site of epithelial damage. For ECRS group, many eosinophils deposited in the basal parts of epithelium or deep in stroma were found to have mostly intact granule, and EET formation was much diminished compared to subepithelial region.

Several studies have reported that NETs are linked to various pathologies, including autoimmune inflammation, infections with thrombosis in sepsis, and allergic diseases^9,28^. Recently, NETs were identified in bronchial biopsies from human asthmatics with a high number of infiltrating neutrophils^17^. However, no extracellular neutrophilic DNA with colocalizing MPO was found in this study. Although, this finding showed similarities with those of the previous publications^25^, we thought that this result was due to the limited or insufficient number of infiltrating neutrophils in our subjects. Further investigations with more tissue samples characterized by a neutrophil-dominant cell type and cell-specific knockout approaches may be required to determine the relative contribution of NETs to the pathogenesis of CRS.

In the present study, we adapted the JESREC scoring system established by a multicentre study in Japan and classified subjects into ECRS and non-ECRS groups using preoperative clinical features of disease side, CT scan, presence of NPs, and peripheral blood eosinophils^4^. Although ECRS is known to more likely be correlated with the presence of tissue eosinophilia, there is no agreed-upon standard system for the definition of tissue eosinophilia worldwide. The most important reason for this disagreement is that the distribution of eosinophils is typically uneven throughout the tissue. If a certain biomarker is to be used for endotyping, the difference in clinical outcome depending on the endotype should be determined. Our study showed that an increased number of EET-releasing eosinophils indicated the presence of a greater disease burden with respect to the blood and eosinophilia as well as increased CT severity score and decreased olfactory function.

These clinical characteristics of EETs may derive from their functional attributes. The expanding DNA traps in the inflamed nasal mucosa contribute to the properties of highly viscous eosinophilic mucin and impairments in its clearance, resulting in long-lasting inflammation and formation of a secondary epithelial barrier defect^15^. As a result, the odorant might be interfered with the passage from nasal cavity into olfactory nerve of olfactory epithelium. Decreased olfaction function in association with EET formation could also suggest a possible role for tissue eosinophilia or eosinophil-associated mucous cytokines that induce cell damage at the neuronal level, likely from inflammatory infiltrate in CRS-associated olfaction dysfunction^29,30^. Further studies, including those evaluating the possible role of EETs in CRS, should be performed to designate new biomarkers that could reflect activated eosinophils with a greater disease burden.

In summary, in this study, we describe the presence and clinical characteristics of EETs representing a novel feature of activated eosinophils infiltrating the nasal mucosa in both ECRS and non-ECRS subjects *in vivo*. The concept of EETs also could have novel implications for potential therapeutic targets, clinical decision-making, and prediction of treatment outcome.

## Supplementary information

Supplementary Figure E1
