# Supplementary material for: Eosinophil extracellular trap formation is closely associated with disease severity in chronic rhinosinusitis regardless of nasal polyp status
Source: Sci Rep. 2019 May 30;9:8061. doi: 10.1038/s41598-019-44627-z (PMC6542829; doi:10.1038/s41598-019-44627-z)

**Eosinophil extracellular trap formation is closely associated with disease severity in chronic rhinosinusitis regardless of nasal polyp status**

Chi Sang Hwang, Sang Chul Park, Hyung-Ju Cho, Dong-Joon Park, Joo-Heon Yoon, Chang-Hoon Kim

ONLINE DATA SUPPLEMENT

**SUPPLEMENTAL INFORMATION**

**Supplementary Figure E1.** A photomicrograph showing that eosinophil distribution is uneven in surgical samples according to CRS phenotype. Unlike in the (A) non-ECRS without NPs and (B) non-ECRS with NPs groups, (C) the ECRS with NPs and (D) ECRS without NPs groups were infiltrated with a higher number of eosinophils, although their numbers varied among individual subjects (haematoxylin and eosin staining, *Scale bar* = 50 µm).


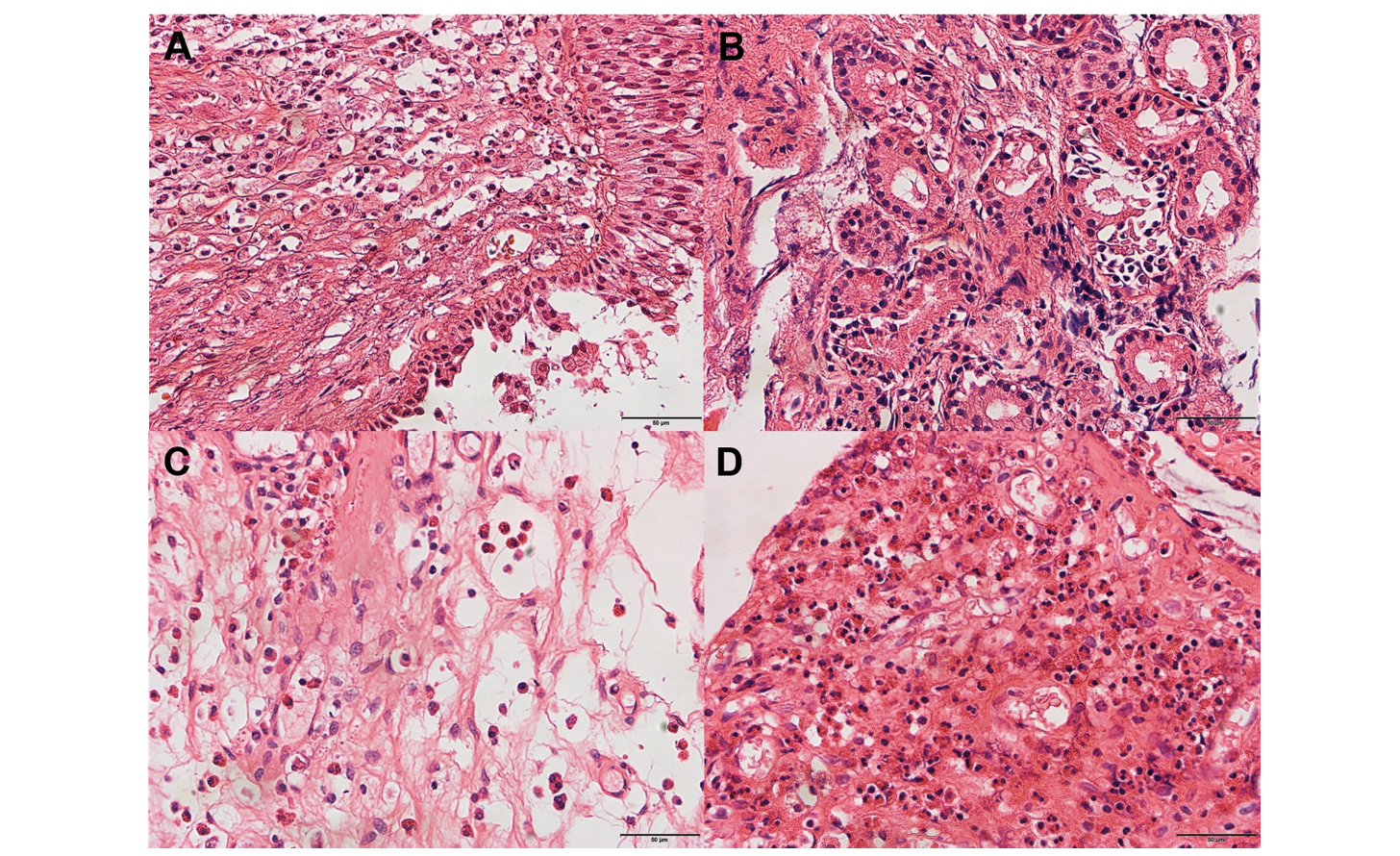

Supplement: Supplementary file 1 — Supplementary Figure E1 [file 41598_2019_44627_MOESM1_ESM.docx]
